# Standardizing Clinical Trials Workflow Representation in UML for International Site Comparison

**DOI:** 10.1371/journal.pone.0013893

**Published:** 2010-11-09

**Authors:** Elias Cesar Araujo de Carvalho, Madhav Kishore Jayanti, Adelia Portero Batilana, Andreia M. O. Kozan, Maria J. Rodrigues, Jatin Shah, Marco R. Loures, Sunita Patil, Philip Payne, Ricardo Pietrobon

**Affiliations:** 1 Research on Research Group, Department of Surgery, Duke University, Durham, North Carolina, United States of America; 2 Biomedical Informatics Program, The Ohio State University College of Medicine, Columbus, Ohio, United States of America; 3 Duke-NUS Graduate Medical School, Singapore, Singapore; 4 Rheumatology, Department of Medicine, State University of Maringá, Maringá, Brazil; 5 Department of Biomedical Informatics, The Ohio State University, Columbus, Ohio, United States of America; 6 Biomedical Informatics Program, The Ohio State University Center for Clinical and Translational Science, Columbus, Ohio, United States of America; 7 Department of Surgery, Duke University Health System, Durham, North Carolina, United States of America; 8 Research on Research Group, Duke University, Durham, United States of America; 9 Kalpavriksha Healthcare and Research, Thane, India; Universidad Peruana Cayetano Heredia, Peru

## Abstract

**Background:**

With the globalization of clinical trials, a growing emphasis has been placed on the standardization of the workflow in order to ensure the reproducibility and reliability of the overall trial. Despite the importance of workflow evaluation, to our knowledge no previous studies have attempted to adapt existing modeling languages to standardize the representation of clinical trials. Unified Modeling Language (UML) is a computational language that can be used to model operational workflow, and a UML profile can be developed to standardize UML models within a given domain. This paper's objective is to develop a UML profile to extend the UML Activity Diagram schema into the clinical trials domain, defining a standard representation for clinical trial workflow diagrams in UML.

**Methods:**

Two Brazilian clinical trial sites in rheumatology and oncology were examined to model their workflow and collect time-motion data. UML modeling was conducted in Eclipse, and a UML profile was developed to incorporate information used in discrete event simulation software.

**Results:**

Ethnographic observation revealed bottlenecks in workflow: these included tasks requiring full commitment of CRCs, transferring notes from paper to computers, deviations from standard operating procedures, and conflicts between different IT systems. Time-motion analysis revealed that nurses' activities took up the most time in the workflow and contained a high frequency of shorter duration activities. Administrative assistants performed more activities near the beginning and end of the workflow. Overall, clinical trial tasks had a greater frequency than clinic routines or other general activities.

**Conclusions:**

This paper describes a method for modeling clinical trial workflow in UML and standardizing these workflow diagrams through a UML profile. In the increasingly global environment of clinical trials, the standardization of workflow modeling is a necessary precursor to conducting a comparative analysis of international clinical trials workflows.

## Introduction

Clinical trials, though historically dominated by the United States and a small subset of countries in North America and Western Europe, are increasingly becoming a global activity with potential implications on health care delivery around the world. Between 1990 and 1999, the number of countries conducting drug research tracked by the Food and Drug Administration [1] rose from 28 to 79 [2], and by one recent estimate, 24 of the fastest 25 growing countries in clinical trials are in emerging, non-traditional areas [3]. A study of industry sponsored phase 3 clinical trials in 2007 revealed that a majority of the sites were outside the United States [4].

Although the globalization of clinical trials brings many potential benefits [3], a major challenge is faced regarding the standardization of clinical trials conducted in different parts of the world; the workflows of clinical trials, as well as the standards of care in different countries may vary so much as to invalidate individual trial results [4]. Ultimately, the relative performance of clinical trials in emerging countries will depend on the internal workflow of these research sites and the establishment of good clinical trials practice guidelines. For example, a 2001 FDA report notes that clinical trial “sponsors have raised concerns regarding the capacity of the institutional review boards in some of the emerging sites to adequately review research according to Good Clinical Practice Guidelines, under the International Conference on Harmonization or FDA standards” [2]. Such variation in clinical practice guidelines among emerging sites has ethical implications, as well as implications on the trial workflow and validity of results. Despite this, little research has been conducted to analyze and/or compare the workflow of clinical trials, let alone those operating across international boundaries [5]. This may, at least partly, be due to the lack of standard computational representation for these workflows, which would facilitate an operational comparison of how clinical trials are being conducted around the world. Additionally, a standard representation would help create more homegeneous clinical trials, which would in turn facilitate the implementation of better meta analyses.

Workflow modeling is an established technique of business process re-engineering, and various studies have assessed its potential in re-engineering organizational processes across various quality measures or goals [6], [7], [8]. For example, workflow modeling in business process re-engineering may be used to identify inefficiencies or opportunities for cost reduction inherent in the sequence of tasks [6]. Yet, the use of workflow modeling in the clinical trials domain is less well established [5]. Few studies have demonstrated the possible use of workflow modeling and analysis towards re-engineering clinical trials [5], [9], [10], and research in this area continues to suffer from the lack of standard representation model. The variety in representation models may extend from the use of different modeling languages to the use of different representations or vocabularies within a single modeling language [9], [10]. For example, different studies may use different modeling languages or symbols to represent the workflow, as well as different semantic phrases to represent the same activity (i.e. “phlebotomy” vs. “drawing blood”). Hence, the need for standardization applies to both the use of a single modeling language, as well as a standard representation to extend the modeling schema into the clinical trials domain.

**Figure 1:**
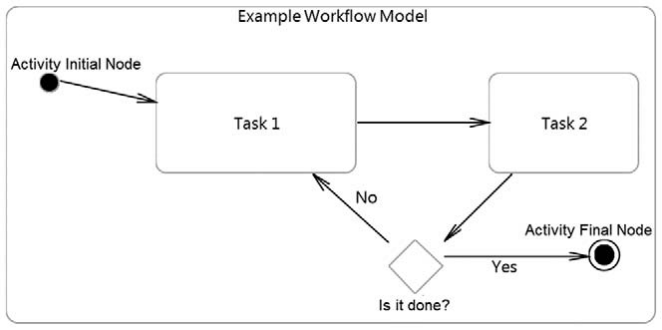
Example workflow model: Workflow begins at an Activity Initial Node and ends at an Activity Final Node; ovals represent actions in the workflow; diamond represents a decision node, where the subsequent direction in the workflow is dependent on a decision.

**Figure 2:**
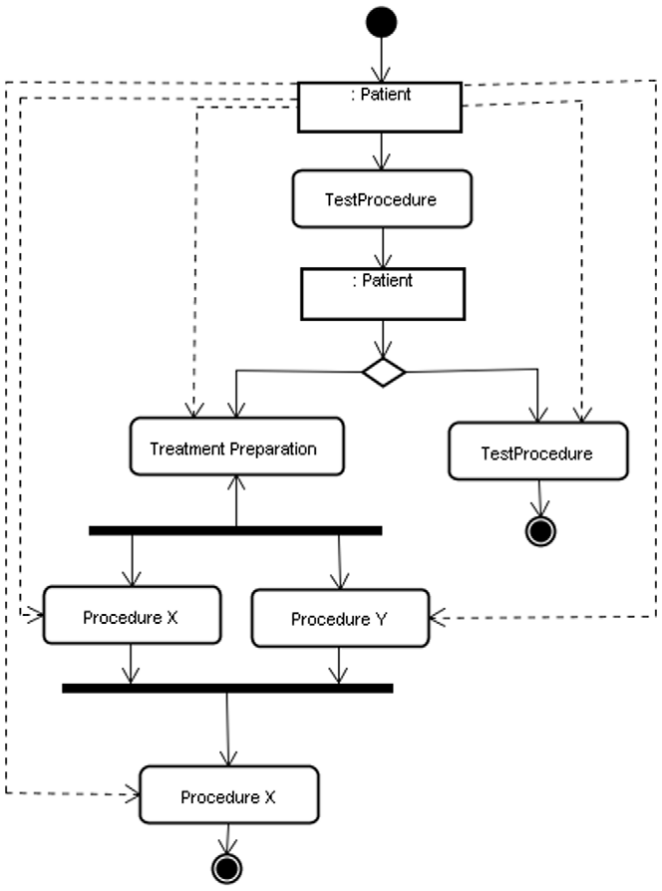
Activity Diagram for a Clinical Process. This activity diagram represents the following activity: the patient is submitted to an activity named “Test Procedure” that generates a result. One of results redirects the flow of activity to an activity called “Refer back to GP” and the activity is finished (filled circle with a border). Another result redirects the flow to a “Treatment Preparation” activity. A black bar with two flow leaving it means that the “Procedure X” and “Procedure Y” are executing in parallel. A black bar with one flow leaving it redirects the flow to the last activity called “Arrange next Appointment” and the filled circle with a border finishs the activity. **Source:** http://citeseerx.ist.psu.edu/viewdoc/download?doi=10.1.1.6.4217&rep=rep1&type=pdf.

Clinical trial modeling in Unified Modeling Language (UML) [11] provides a potential solution to some of these problems and can serve as a standard format for workflow modeling. (Figure 1 provides an example of a workflow model in UML) UML allows the detailed description of organizational processes in a so-called Activity Diagram (AD), which can be annotated with data to support process analysis [12], [13]. (Figure 2 depicts a real world example of an activity diagram representing a clinical process.) Various studies have established the use of the UML activity diagram in modeling of business processes [13], and some have even demonstrated its use in the healthcare domain [14], [15], [16]. In order to define a standard representation for UML models within a domain like clinical trial, one can formulate a UML profile [17], which enables independent developers to generate standardized UML models at different sites [18], [19]. The UML profile can be loaded into a UML developing environment in order to apply a standard set of data tags (attributes) to a workflow model; for example, a UML profile defining a standard for colonoscopy workflow might include a standard set of data attributes including the type of endoscope being used, the names of physicians or nurses, the type of sedation being used, and the duration of the procedure. To this end, a UML profile for clinical trial workflow might specify attributes which facilitate the gathering of data for a time-motion study [20] to allow comparisons of efficiency in line with the NIH roadmap goal of re-engineering clinical research [21].

Given the rapid global expansion of clinical trials, the creation of standard nomenclature for clinical trial workflow representation can facilitate the analysis and comparison of workflows across international sites. A standardized workflow representation might also enable analyses of efficiency and cost, thereby allowing researchers to shorten the length of a research study and expedite the incorporation of sound research results into the healthcare system. Similarly, the development of standard workflow diagrams can aid in the process of establishing good clinical trial practice guidelines across international sites participating in a given study.

The objective of this study was to design a UML profile to extend the UML AD schema into the clinical trial domain, thereby defining a standard representation for clinical trial workflow diagrams modeled in UML at different sites. In designing this profile, we paid particular attention to attributes which may lend a description of time, distribution, efficiency, and, ultimately, cost.

## Methods

The study was approved by the local Institutional Review Board at State University of Maringa, Brazil (Comitê Permanente de Ética em Pesquisa Envolvendo Seres Humanos – COPEP - da Universidade Estadual de Maringá - UEM). Verbal informed consent was obtained as per guidance provided by the IRB, since this is an observational study where no personal information was recorded, thus anonymizing the study data. We evaluated the workflow of clinical trials conducted at two private clinics, one each in the cities of Maringa and Rio de Janeiro, Brazil. To protect subject confidentiality as well as intellectual property of the companies conducting the clinical trials, no clinical trial data was accessed by workflow modelers. Subjects were anonymous to the research team.

### Study sample

The clinical trial sites were evaluated for a total of 53-clinic hours, involving clinical trials related to rheumatology and oncology. At both sites, a small research team was present with a single clinical research coordinator (CRC) managing 5 to 6 clinical trials. During clinic visits, we conducted a series of ethnographic observations, also performing interviews with the CRC, principal investigator, and other staff direct or indirectly involved with clinical trial activities. All notes and interviews were focused on workflow issues, their variations across different circumstances, and subject's perceptions about their effectiveness and points of failure. Observations were recorded in field notes, which were later transcribed and analyzed to create a list of workflow tasks.

### Ethnographic study: interviews and time motions studies

#### Observation Categories

Observers [EC, AB] compiled an overall list of activities from summarization of the original ethnographic study and findings from previous studies, and then subsequently documented study activities by choosing descriptors from this list [10], [22]. These activities were hierarchically classified into major and minor activities based on consensus among the two researchers collecting the data (EC, AB), thus facilitating data collection.

#### Data Entry

Observers [EC, AB] recorded data in a laptop using a MySQL database run locally from a Web browser interface specially designed for this study. Beginning and end times were recorded for each task. For each activity, observers recorded an observation session ID, observer ID, CRC ID, and time stamp measured to the nearest second. When a CRC was engaged in multiple activities at the same time, such as “Asking the patient how he is feeling” and “Recording in the chart,” the observer ranked one profile as a primary activity and the other as a secondary activity. If a CRC switched from one activity to another in rapid succession, these activities were recorded in sequence. Long transitional periods between separate activities were logged as a separate activity under the descriptor “other,” while short transitional periods associated, for example, with the time elapsed between “taking the pulse” and “recording in the chart”, were logged as part of the second activity's total duration [22].

### UML modeling

As described in the introduction, Unified Modeling Language (UML) is a computational language that can be used to represent workflows of operational processes [11]. Accordingly, UML can be used to model the workflow of businesses, procedures, or any healthcare activity. In this study, we have used UML to model the operational workflow of clinical trials.

All workflows in this study were modeled in UML 2.0 ADs via the UML2 plugin [23] for Eclipse[24]. In our manuscript the term “UML” is often used in lieu of “UML2” to refer to diagrams created via this UML2 plugin. Events in the workflow are represented as oval-shaped structures called “Opaque Actions”, related to one another by transition arrows or “Control Flows”. “Decision Nodes” are diamond-shaped elements that represent forks in the workflow where the outgoing path depends on the outcome of a decision (i.e. has the patient been consulted by a doctor?). The overall workflow begins at an “Activity Initial Node” and ends at an “Activity Final Node.” All workflows confirm to UML 2.0 standards as implemented in Eclipse.

**Figure 3:**
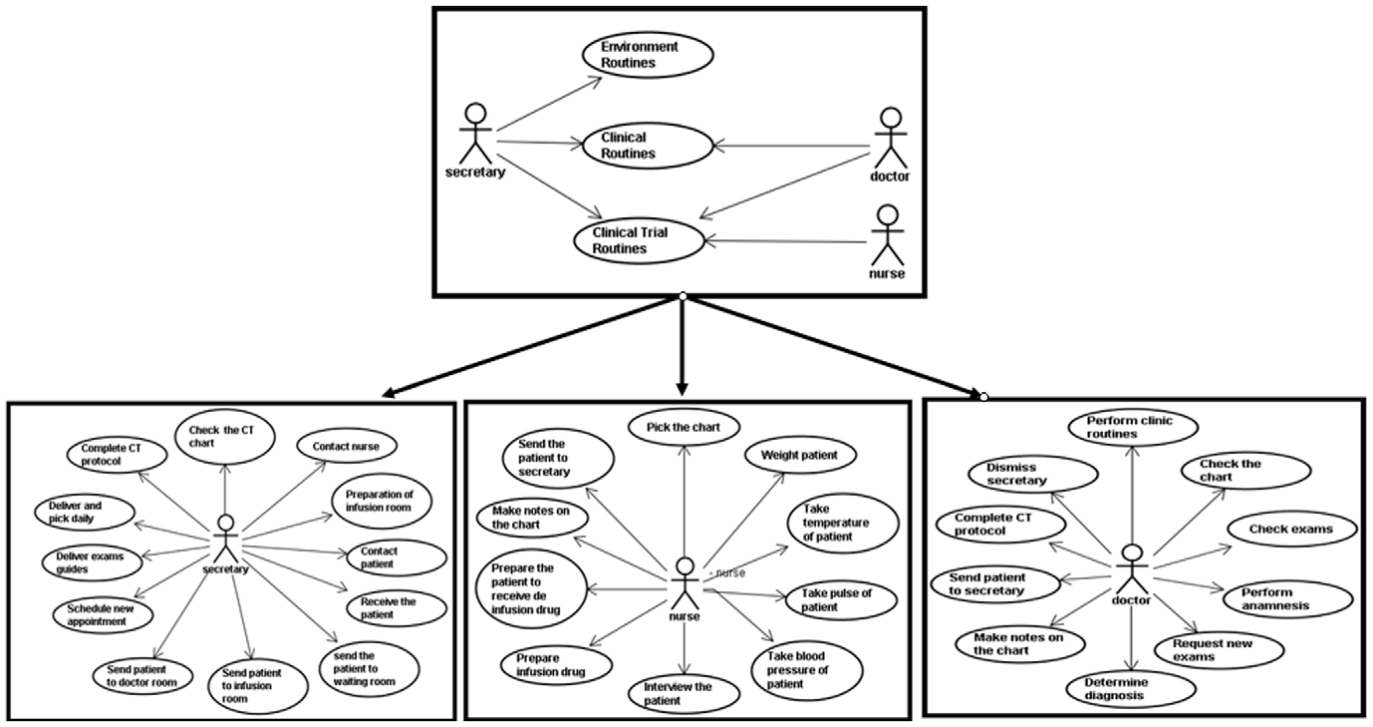
A global use case to specific actor use cases.

In order to annotate the workflow, a UML profile (S 2) was designed to incorporate all information from the ethnographic and time-motion studies into the AD based on a use-case (fig 3) using criteria defined in Table 1. The use-case describes a scenario showing the functionality of the system from the view of the user [11]. Meanwhile, the UML profile includes “stereotypes” or grouped sets of attributes, which apply time, distribution, and cost information to the workflow. The attributes were devised to annotate the activity diagram (File S3) with the quantities of information included in simulation software packages such as AnyLogic [25] or Arena Simulation Software [26].

**Table 1:** Criteria to build a use case for simulation.

**Goal**	The goal of this use case is to drive the design of a UML profile to extend Activity Diagrams (AD). This extension will allow researchers to upload UML ADs directly into computer simulation software as well as establish workflow comparison across different clinical trials.
**Summary**	The use case presents the creation of a UML AD with a description of its time as well as distribution components designed to be compliant with the required information for a computer simulation.
**Actors**	The UML modeler and the computer simulation specialist, although very often these two actors will be represented by the same person.
**Pre-conditions**	A researcher conducts an ethnographic study evaluating the workflow of a clinical trial site, resulting in a list of activities placed in the most common sequential order. A time motion study is then conducted to assign average completion times for each of the activities.
**Triggers**	The case is initiated by the completion of the data collection stage of the ethnographic and time motion studies. These stages provide the data that will populate the UML AD.
**Basic course of events**	A UML activity diagram is created with the tasks in sequence as usually performed in a regular UML AD. These tasks are then tagged with time data including multiple distribution parameters. Distribution parameters are also attached to different random decision nodes. The UML AD is then uploaded to a computer simulation package used for discrete event simulation, and then converted to a simulation model
**Post-conditions**	The UML AD is converted into a computer simulation model, and any modifications made during the calculation of the analytical solution to the model are automatically translated into the UML AD.

The profile can be applied to any AD created in Eclipse, thereby standardizing the types of attributes applied to elements of the workflow. For example, a hypothetical “time” stereotype in a profile, containing duration and delay information, might be applied to events in the workflow in order to annotate the diagram with this data. Attributes in our profile which can be applied to Opaque Actions include measures of duration, delay, fail rate, rework rate, communication rate, and the units of measurement used; attributes which can be applied to Decision Nodes include measures of distribution, including the beta, continuous, discrete, Erlang, exponential, gamma, Johnson, lognormal, normal, Poisson, triangular, uniform, and Weibull distributions (see Table 2 for details), in accordance with the statistical distribution information included in Arena Simulation Software [27].

**Table 2:** Details on distribution parameters.

Beta (Beta, Alpha)	Beta (β) and Alpha (α) specified as positive real numbers.
Continuous (P1, V1, …)	P1 is a Pair of cumulative probabilities and V1 is an associated value.
Discrete(P1, V1, …)	P1 is a Pair of cumulative probabilities and V1 is an associated value.
Erlang(ExpoMean, k)	: ExpoMean are distributed exponential random variables and k is the number of exponential random variables.
Exponential(Mean)	The mean (β) specified as a positive real number.
Gamma(Beta, Alpha)	Shape parameter (α) and scale parameter (β) specified as positive real values.
Johnson(Gamma, Delta, Lambda, Xi)	Gamma shape parameter (γ), Delta shape parameter (δ>0), Lambda scale parameter (λ), and Xi location parameter (ξ).
Lognormal(LogMean, LogStd)	Mean LogMean and standard deviation LogStd of the lognormal random variable. Both LogMean and LogStd must be specified as strictly positive real numbers.
Normal(Mean, StdDev)	The mean (µ) specified as a real number and standard deviation (σ) specified as a positive real number.
Poisson(Mean)	The mean (λ) specified as a positive real number.
Triangular(Min, Mode, Max)	The minimum (a), mode (m), and maximum (b) values for the distribution specified as real numbers with a<m<b.
Uniform(Min, Max)	The minimum (a) and maximum (b) values for the distribution specified as real numbers with a<b.
Weibull (Beta,Alpha)	Shape parameter (α) and scale parameter (β) specified as positive real values.

*extracted from Arena User's Guide, 2007.

## Results

### Ethnographic notes on tasks

The ethnographic observations generated a list of tasks that are listed and classified hierarchically by the following categories: environment, clinical trial, clinical routines (File S1). Recurring patterns observed across tasks that impaired workflow included (a) CRCs in charge of tasks that required full commitment from them (e.g., computerized tomography) resulted in long idle times where the research coordinator could not accomplish other activities (b) the transmission of notes from paper to electronic data capture systems frequently resulted in activities that required extreme attention and therefore were more prone to errors that could go undetected (c) lack of use of standard operating procedures frequently led to rework in workflow, since the first attempt to execute an activity was accompanied by error or a missing step such as when a CRC forgot a portion of the paper-based medical record while coming to a subject evaluation and (d) lack of integration across different information technology systems, such as the electronic data capture system and the adverse event reporting system being from different software packages.

### Time and motion

**Figure 4:**
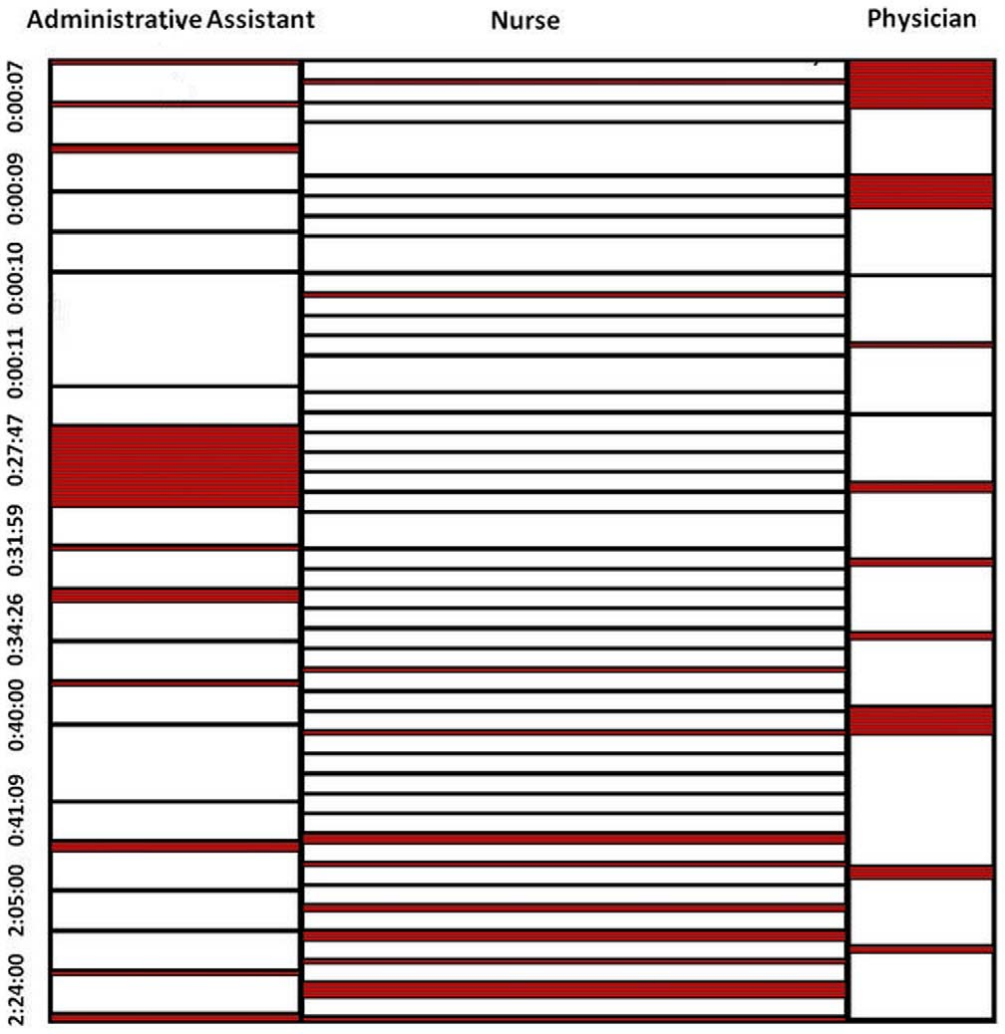
Summary of time & motion data for actors.

Time & motion data for different hierarchical activities are summarized through a mosaic chart where the width of the bar is scaled to the proportion of the time required to complete the task, comparing categories for physicians, nurses, and administrative assistants (figure 4).

**Figure 5:**
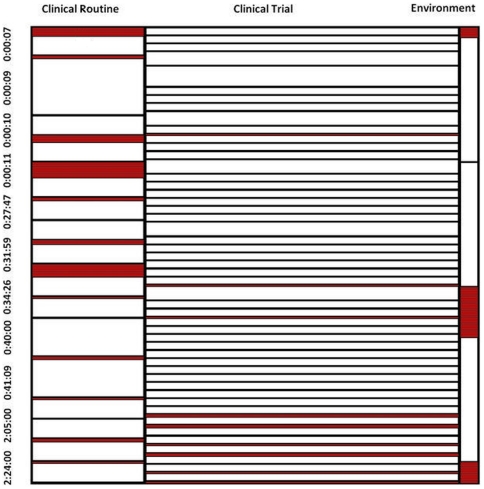
Summarization of time & motion data for task activities.

In Figure 4, white color indicates that the actors were busy performing the task while red color indicates idle time or gaps between tasks. The box width represents the proportion of tasks performed by each of the actors amongst all the tasks. Box height represents the time required to complete the task, and timestamps along the vertical axis represent elapsed time in the workflow. Physicians perform tasks consistently with shorter idle times, but towards the end of the workflow they have relatively longer idle time as compared with others. Physicians also have relatively a smaller number of tasks compared to others, but they tend to require a longer time for completion. At the beginning of the workflow nurses have a smaller frequency of tasks and more idle time but during the course of the workflow this frequency increases, resulting in very short idle times compared to others. Nurses have a higher frequency of tasks as compared to others, with time to completion being shorter and consistent throughout the workflow. Finally, administrative assistants have fewer gaps between their tasks at the start and the end of workflow, with but has relatively longer idle time in the middle of the workflow. Their task frequency is somewhere in the middle between nurses and physicians, with the time required for task completion also being in middle range. Figure 5, represents a mosaic chart comparing tasks related to clinic routines, activities specific to the clinical trial, and general tasks.

Tasks specific to the clinical trial present a few short gaps between them at the start of the workflow, but throughout the workflow period these tasks are performed in a clustered pattern. These tasks have a greater frequency compared to tasks labeled as routine or general, also having a shorter duration as compared to others. Clinical routine tasks are initially performed at consistent intervals, but idle times become greater towards the middle and end of the workflow. Finally, tasks labeled as related to the environment had a low frequency, but took long times for completion.

### UML profile

**Figure 6:**
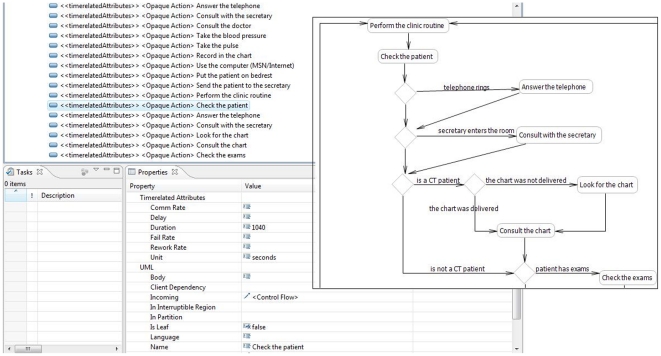
Portion of the Activity Diagram created with the UML Profile, highlighting data annotation for “Check the patient”.

We furnish the link for a UML profile designed in Eclipse to incorporate additional characteristics required for workflow modeling in clinical research. (File S2) It includes measures of time and distribution, in accordance with the types of information used in discrete event simulation models. The profile makes use of “stereotypes,” which are grouped sets of attributes; the attributes, here, are technically “child” attributes, meaning that they hierarchically belong to the “parent” stereotype. In the profile we've created, the “Time-related attributes” stereotype includes child property attributes of delay, fail rate, rework rate, communication rate, duration, and units, which is defined under an enumeration literal to take on a value of “seconds” or “minutes.” The “Time-related attributes” stereotype can be applied to Opaque Actions representing events in an AD. Figure 6 displays the annotation of the Opaque Action, “Check the patient,” with the “Time-related attributes” stereotype.

Additional stereotypes were created for each of the distribution types (beta, continuous, discrete, etc.) previously described, with each distribution stereotype containing the appropriate child attributes corresponding to the mathematical distribution (i.e. the beta distribution stereotype contains child property attributes for distribution parameters β and α). Each distribution stereotype can be applied to decision nodes in an AD in order to annotate these nodes with data according to a specific distribution. Although we did not make use of any “generalizations” in our UML profile, the “generalization” feature allows for the specification of hierarchical relationships between stereotypes, such that a more specific stereotype inherits all attributes of a more general stereotype. A child generalization created for a given sub-stereotype can refer to a super-stereotype, from which the sub-stereotype will inherit all attributes [18].

## Discussion

To our knowledge, this is the first study evaluating the use of UML activity diagrams to standardize the representation of workflow in the clinical trial domain, thus extending previous applications of UML in healthcare operations [14], [15], [16]. Our main findings included ethnographic observations of patterns of activities which caused workflow problems, and time-motion analysis information regarding the relative duration of activities performed by different actors in the workflow. Specifically, workflow problems occurred with tasks requiring the full commitment of clinical trial team, such that they could not multi-task, the transfer of information from paper to electronic systems, occasional deviations from standard operating protocols, and idle time resulting from a lack of integration between different IT systems. Our time-motion analysis also revealed the following potential areas for intervention in the workflow to improve efficiency: first, physician downtime appears to be greatest near the beginning of the workflow and, accordingly, efficiency can be increased by other actors performing all necessary tasks prior to the physician entering the workflow, such that the physician can perform tasks in an uninterrupted flow. Second, the administrative assistants appear to experience the greatest downtime during the middle of the workflow; accordingly, this would be an appropriate time to take care of all environment or clinic routine tasks unrelated to the clinical trial, in order to ensure the smooth running of clinical trial activities. Third, the nurses appear to be busy throughout the workflow, and so it appears that making sure that their participation in the workflow runs efficiently at all times may have the greatest overall impact on the efficiency of the clinical trial workflow. Overall, clinical trial tasks were the most prevalent tasks on the overall workflow than either clinic routines or other general activities.

Though several studies [5], [9], [10] have examined workflow modeling of clinical trials, they suffer from the lack of a standard representation model. To the best of our knowledge, this is the first study to use UML for modeling the operational workflow of clinical trials. Furthermore, we have incorporated a time-motion study into our workflow analysis via a UML profile. The UML profile also serves to create a standard representation for clinical trials workflow ADs, thereby facilitating comparison.

In the UML profile, stereotypes provide a convenient means for creating a standard set of attributes for UML models. However, the UML profile would be a more effective tool if given the ability to restrict the semantics of UML diagrams. For example, if we could have used our UML profile to limit the potential descriptors of Opaque Actions in UML ADs to only those terms from a standardized list of clinical trial activities, we could have effectively promoted the use of a single, standardized vocabulary in the creation of UML ADs modeling clinical trial workflow. With the current profile, however, workflow models created at different sites might conceivably use different descriptors for analogous or equivalent activities, thus hindering comparative analysis of these models.

Limitations of our study which could be subjects for future research are as follows: first, we only evaluated a limited number of sites in rheumatology and oncology - future studies should examine more types of research groups with different workflows. Second, though we have used a use case towards the goal of directly importing workflow models into discrete event simulation software, to our knowledge there is currently no import function of this type available in any software. Future research should develop interfaces with existing packages so that importing UML activity diagrams into simulation software can be formally tested. Third, though we dealt with standardizing the data annotation for UML workflow models of clinical trials, we did not address the standardization of terminologies or vocabularies used in these workflow models. To reiterate, the UML profile would greatly benefit from the ability to restrict the semantics of UML diagrams.

Further, we also did not generate an international comparison of clinical trials workflows using our profile as this was beyond the scope of our study, and thus we have not explicitly demonstrated the reengineering of a clinical research group based on information gathered from our UML workflow analysis. Future works should utilize the UML profile towards comparing workflows of clinical research groups in different countries and demonstrate the use of this analysis towards actually reengineering the workflow of a clinical research group. This reengineering process might then be evaluated for its effectiveness in improving clinical trials workflow across various quality measures or goals.

We should also note that while this paper makes progress towards developing a standardized computational representation for clinical trial workflow, much work remains to be done in order to establish a true standard representation. Our work is limited to the particular clinical trials we have studied, and many more should be examined before a true standard can be developed. Future research might expand this project through examining more types of clinical trials and developing a standard terminology for the processes associated with these clinical trials.

In conclusion, this paper describes a method for modeling clinical trials workflows in UML Activity Diagrams and standardizing these workflow diagrams through a UML profile. The model we've created demonstrates the process of building a standard clinical trial model in UML and annotating it with time-motion data. In the increasingly global environment of clinical trials, the standardization of workflow modeling is a necessary precursor to conducting a comparative analysis of international clinical trials workflows. Future research might use this standardized workflow representation to generate workflow diagrams of clinical trials in emerging countries and compare these to workflow diagrams of clinical trials in the US.

## Supporting Information

File S1 Tasks during ethnographic observation and respective actors.
(0.11 MB DOC)

File S2 XMI code for UML Profile for Clinical Research available.
(0.03 MB DOC)

File S3 Activity Diagram created on Eclipse using UML2.
(0.06 MB DOC)
