# Supplementary material for: Standardizing Clinical Trials Workflow Representation in UML for International Site Comparison
Source: PLoS One. 2010 Nov 9;5(11):e13893. doi: 10.1371/journal.pone.0013893 (PMC2976698; doi:10.1371/journal.pone.0013893)
Supplement: File S1 — Tasks during ethnographic observation and respective actors. (0.11 MB DOC) [file pone.0013893.s001.doc]

## Supporting information

**S1: Tasks during ethnographic observation and respective actors**

| **Actor** | **Category** | **Task** | **Time 1 (sec)** | **Time 2 (sec)** | **Time 3 (sec)** |
| --- | --- | --- | --- | --- | --- |
| Secretary | Environment | Open room | 00:00:15 | 00:00:17 | 00:00:18 |
| Secretary | Environment | Turn on the lights | 00:00:20 | 00:00:18 | 00:00:15 |
| Secretary | Environment | Turn on the computers | 00:02:15 | 00:03:05 | 00:03:28 |
| Secretary | Clinic Routine | Check the chart | 00:22:26 | 00:28:30 | 00:32:26 |
| Secretary | Clinical Trial | Prepare the infusion room | 00:12:43 | 00:14:43 | 00:10:00 |
| Secretary | Clinic Routine | Perform the office routines | 00:30:44 | 00:28:12 | 00:37:00 |
| Secretary | Clinic Routine | Check the patient | 00:12:35 | 00:11:50 | 00:10:22 |
| Secretary | Clinic Routine | Call the nurse | 00:00:20 | 00:00:22 | 00:00:21 |
| Secretary | Clinic Routine | Send patient to waiting room and wait the nurse | 00:00:20 | 00:00:20 | 00:00:19 |
| Secretary | Clinic Routine | Send patient to the waiting room | 00:00:15 | 00:00:12 | 00:00:14 |
| Secretary | Clinical Trial | Deliver the chart | 00:00:08 | 00:00:09 | 00:00:10 |
| Secretary | Clinical Trial | Send patient to the nurse | 00:00:10 | 00:00:10 | 00:00:12 |
| Nurse | Clinical Trial | Weigh patient | 00:00:28 | 00:00:30 | 00:00:32 |
| Nurse | Clinical Trial | Ask the patient to sit on the table | 00:00:12 | 00:00:10 | 00:00:17 |
| Nurse | Clinical Trial | Place the thermometer in the patient's mouth | 00:00:10 | 00:00:15 | 00:00:10 |
| Nurse | Clinical Trial | Take the blood pressure | 00:00:50 | 00:01:00 | 00:00:45 |
| Nurse | Clinical Trial | Take the pulse | 00:00:30 | 00:00:28 | 00:00:35 |
| Nurse | Clinical Trial | Remove the thermometer and record the temperature | 00:00:55 | 00:00:45 | 00:00:50 |
| Nurse | Clinical Trial | Tell the patient to lay on the table | 00:00:05 | 00:00:07 | 00:00:09 |
| Nurse | Clinical Trial | Question the patient | 00:02:50 | 00:02:10 | 00:02:35 |
| Nurse | Clinical Trial | Get the scissors | 00:00:15 | 00:00:20 | 00:00:18 |
| Nurse | Clinical Trial | Get the medication box with scissors | 00:00:17 | 00:00:20 | 00:00:16 |
| Nurse | Clinical Trial | Open the medication out of the box | 00:00:35 | 00:00:35 | 00:00:55 |
| Nurse | Clinical Trial | Take the medications out of the box | 00:00:30 | 00:00:34 | 00:00:32 |
| Nurse | Clinical Trial | Take the syringe out of the box | 00:00:12 | 00:00:10 | 00:00:15 |
| Nurse | Clinical Trial | Throw the box and the packaging in the trash | 00:00:10 | 00:00:10 | 00:00:10 |
| Nurse | Clinical Trial | Open medication bottles | 00:01:15 | 00:01:30 | 00:01:10 |
| Nurse | Clinical Trial | Mix the medictions with the syringe | 00:01:20 | 00:01:30 | 00:01:40 |
| Nurse | Clinical Trial | Take 20ml of saline from the saline bag | 00:00:42 | 00:00:37 | 00:00:47 |
| Nurse | Clinical Trial | Put 20 ml of medication into the saline bag | 00:00:30 | 00:00:28 | 00:00:30 |
| Nurse | Clinical Trial | Get the saline bag from the refrigerator | 00:00:23 | 00:00:30 | 00:00:27 |
| Nurse | Clinical Trial | Clean the saline bag | 00:00:51 | 00:00:55 | 00:00:58 |
| Nurse | Clinical Trial | Perform hand hygiene | 00:01:00 | 00:01:02 | 00:01:10 |
| Nurse | Clinical Trial | Open the infusion kit (needle, cotton) | 00:00:38 | 00:00:47 | 00:00:35 |
| Nurse | Clinical Trial | Perform hand hygiene | 00:01:05 | 00:01:12 | 00:01:15 |
| Nurse | Clinical Trial | Clean the saline again | 00:00:37 | 00:00:45 | 00:00:50 |
| Nurse | Clinical Trial | Throw the packaging in the trash | 00:00:10 | 00:00:14 | 00:00:10 |
| Nurse | Clinical Trial | Find patient´s vein | 00:02:40 | 00:02:10 | 00:02:20 |
| Nurse | Clinical Trial | Take the scissors and cut patches | 00:01:20 | 00:01:30 | 00:01:50 |
| Nurse | Clinical Trial | Put the needle in the saline | 00:00:15 | 00:00:10 | 00:00:12 |
| Nurse | Clinical Trial | Clean tha patient´s arm | 00:00:20 | 00:00:30 | 00:00:33 |
| Nurse | Clinical Trial | Tie the tourniquet on the patient´s arm | 00:00:20 | 00:00:30 | 00:00:30 |
| Nurse | Clinical Trial | Remove the tourniquet from the patient´s arm | 00:00:20 | 00:00:15 | 00:00:17 |
| Nurse | Clinical Trial | Change arms | 00:00:20 | 00:00:22 | 00:00:25 |
| Nurse | Clinical Trial | Get the scissors | 00:00:20 | 00:00:25 | 00:00:15 |
| Nurse | Clinical Trial | Out a patch | 00:01:05 | 00:01:15 | 00:01:10 |
| Nurse | Clinical Trial | Insert the needle in the patient´s arm | 00:00:20 | 00:00:10 | 00:00:12 |
| Nurse | Clinical Trial | Ask the patient how is he feeling | 00:00:30 | 00:00:25 | 00:00:20 |
| Nurse | Clinical Trial | Make notes in the chart | 00:01:40 | 00:01:10 | 00:01:30 |
| Nurse | Clinical Trial | Sit at the desk and use the coputer (MSN) | 00:28:00 | 00:18:00 | 00:22:00 |
| Nurse | Clinical Trial | Perform the infusion | 02:00:00 | 02:10:00 | 02:05:00 |
| Nurse | Clinical Trial | Take the blood pressure | 00:01:30 | 00:01:20 | 00:01:44 |
| Nurse | Clinical Trial | Take the pulse | 00:01:23 | 00:01:20 | 00:01:30 |
| Nurse | Clinical Trial | Take the temperature | 00:02:14 | 00:02:10 | 00:02:20 |
| Nurse | Clinical Trial | Record in the chart | 00:09:00 | 00:12:00 | 00:10:00 |
| Nurse | Clinical Trial | Use the computer (MSN/Internet) | 00:10:00 | 00:18:00 | 00:15:00 |
| Nurse | Clinical Trial | Put the patient on bedrest | 00:00:20 | 00:00:30 | 00:00:27 |
| Nurse | Clinical Trial | Send the patient to the secretary | 00:00:45 | 00:01:08 | 00:00:55 |
| Secretary | Clinic Routine | Check the patient | 00:12:35 | 00:11:50 | 00:10:22 |
| Secretary | Clinic Routine | Send patient to waiting room and wait the doctor | 00:00:20 | 00:00:20 | 00:00:19 |
| Secretary | Clinic Routine | Send patient to the doctor | 00:00:20 | 00:00:21 | 00:00:19 |
| Doctor | Clinic Routine | Perform the clinic routine | 02:20:00 | 02:30:00 | 02:22:00 |
| Doctor | Clinic Routine | Check the patient | 00:20:00 | 00:17:00 | 00:15:00 |
| Doctor | Clinical Trial | Look for the chart | 00:01:00 | 00:01:20 | 00:01:10 |
| Doctor | Clinical Trial | Consult the chart | 00:02:00 | 00:02:30 | 00:02:34 |
| Doctor | Clinic Routine | Check the exams | 00:01:10 | 00:01:00 | 00:01:15 |
| Doctor | Clinic Routine | Perform anamnesis | 00:20:00 | 00:15:00 | 00:17:00 |
| Doctor | Clinic Routine | Determine the diagnosis | 00:01:00 | 00:01:10 | 00:02:00 |
| Doctor | Clinic Routine | Request exams | 00:01:15 | 00:01:35 | 00:01:05 |
| Doctor | Clinical Trial | Include the data in the chart | 00:02:00 | 00:01:20 | 00:01:55 |
| Doctor | Clinic Routine | Send patient to the secretary | 00:00:30 | 00:00:25 | 00:00:33 |
| Secretary | Clinic Routine | Schedule a new appointment | 00:45:09 | 00:40:10 | 00:38:09 |
| Secretary | Clinic Routine | Deliver the exam guides | 00:10:04 | 00:11:04 | 00:13:04 |
| Secretary | Clinical Trial | Check information from the previous day | 00:02:03 | 00:01:26 | 00:01:50 |
| Secretary | Clinical Trial | Deliver new daily information | 00:01:05 | 00:02:20 | 00:01:25 |
| Secretary | Clinic Routine | Release the patient | 00:06:06 | 00:07:13 | 00:06:50 |
| Secretary | Clinical Trial | Complete protocol documents | 00:35:09 | 00:28:09 | 00:40:00 |
| Doctor | Clinical Trial | Complete protocol documents | 00:35:00 | 00:40:00 | 00:45:00 |
| Doctor | Clinic Routine | Dismiss the secretary | 00:01:30 | 00:01:18 | 00:01:44 |
