# Supplementary material for: Standardizing Clinical Trials Workflow Representation in UML for International Site Comparison
Source: PLoS One. 2010 Nov 9;5(11):e13893. doi: 10.1371/journal.pone.0013893 (PMC2976698; doi:10.1371/journal.pone.0013893)
Supplement: File S2 — XMI code for UML Profile for Clinical Research available. (0.03 MB DOC) [file pone.0013893.s002.doc]

## Supporting information

**S2: XMI code for UML Profile for Clinical Research available at**[**https://sourceforge.net/projects/uml2clinical/**](https://sourceforge.net/projects/uml2clinical/)
