# Supplementary figures and images for: Standardizing Clinical Trials Workflow Representation in UML for International Site Comparison
Source: PLoS One. 2010 Nov 9;5(11):e13893. doi: 10.1371/journal.pone.0013893 (PMC2976698; doi:10.1371/journal.pone.0013893)

## Supporting information

**S 3: Activity Diagram created on Eclipse using UML2**


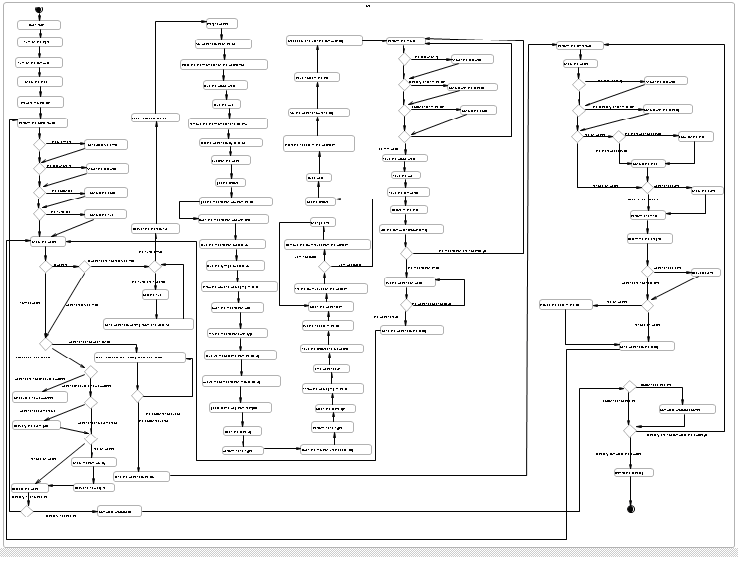

Supplement: File S3 — Activity Diagram created on Eclipse using UML2. (0.06 MB DOC) [file pone.0013893.s003.doc]
